# New Actions on Actionable Mutations in Lung Cancers

**DOI:** 10.3390/cancers15112917

**Published:** 2023-05-26

**Authors:** Xiuning Le, Yasir Y. Elamin, Jianjun Zhang

**Affiliations:** 1 Department of Thoracic/Head and Neck Medical Oncology, The University of Texas MD Anderson Cancer Center, Houston, TX 77030, USA; xle1@mdanderson.org (X.L.); yyelamin@mdanderson.org (Y.Y.E.); 2 Department of Genomic Medicine, The University of Texas MD Anderson Cancer Center, 1515 Holcombe Blvd, Houston, TX 77030, USA

Actionable mutations refer to DNA alterations that, if detected, would be expected to affect patients’ response to treatments [1]. Among these actionable mutations, the most clinically impactful ones can be targeted with specific drugs or therapies, known as targetable mutations. In non-small cell lung cancer (NSCLC), nine oncodriver genes (EGFR, ERBB2 (HER2), ALK, ROS1, RET, NTRK, MET, BRAF, and KRAS) have been found to carry targetable mutations. Therapies targeting these mutations include small-molecule tyrosine kinase inhibitors (TKIs), monoclonal antibodies, and antibody-drug conjugates. Targeted therapies for these mutations have shown significant improvements in response rates, progression-free survival (PFS), and overall survival (OS), thereby revolutionizing the treatment of NSCLC. However, not all patients with targetable mutations respond equally to targeted therapies, and resistance eventually develops. Ongoing research aims to continue improving the outcomes for NSCLC patients with actionable mutations. This Special Issue on “Actionable Mutations in Lung Cancer” includes a collection of studies that address various vital questions, as highlighted in the following examples.

One critical area of active investigation is determining which subgroup of patients will benefit from targeted therapy alone versus intensified therapy. The combination of TKIs with different therapeutic modalities, including chemotherapy [2], radiation therapy [3], and anti-angiogenesis [4], has been tested as an important approach to overcome intra-tumor heterogeneity [5,6,7] and/or achieve a synergistic effect to improve patient outcomes in oncodriver mutant NSCLC. Several publications in this Special Issue aim to address this question through retrospective analyses of published clinical trials and real-world data. In a meta-analysis by Xue et al., the efficacy and safety of various combination treatments in the first-line setting for metastatic EGFR-mutant NSCLC were compared [8]. The study found that TKI combined with antiangiogenic therapy, chemotherapy, or radiation achieved superior PFS compared to TKI alone, with radiation providing the most additional benefits. Moreover, a combination with pemetrexed/carboplatin chemotherapy or radiation was associated with superior OS compared to TKI alone. However, these additional benefits from combination therapy were accompanied by higher therapy-associated toxicities. Therefore, the decision-making process between oncologists and patients should consider both efficacy and treatment-specific toxicities when deciding between TKI alone and combination therapy.

Patients with inferior outcomes with the current standard-of-care targeted therapy are more likely to benefit from therapy intensification. For example, in an extensive real-world analysis of 356 patients with advanced EGFR-mutant NSCLC by Le et al. in this issue, co-occurring TP53 mutations were confirmed to be associated with inferior PFS and OS [9]. Since EGFR-mutant NSCLC with co-occurring TP53 mutations has a poor prognosis with standard-of-care TKIs, the next step will be to test whether these patients benefit more from the intensification of first-line therapy.

NSCLC patients with targetable mutations generally benefit less from immune checkpoint blockade (ICB), with possibly the exception of BRAF mutations and KRAS mutations [10,11]. In this Special Issue, Hong et al. specifically evaluated the efficacy of adding anti-PD1/L1 therapy to platinum-based chemotherapy in TKI-resistant EGFR-mutant NSCLC using a relatively large real-world cohort (*n* = 178). The study found that immunotherapy adds limited benefit to platinum doublets regardless of PD-L1 levels [12]. In addition, it is known that mutations in cancer genes such as STK-11 or KEAP1 are associated with ICB benefit independent of PD-L1 and tumor mutation burden [13,14], highlighting the critical potential of these genomic features as predictive biomarkers for ICB treatment. Three studies in this Special Issue attempted to identify novel genomic features as predictive markers to guide ICB-based therapy. Zhang et al. reported that mutations in HSPG2 were associated with benefits from ICB in melanoma and NSCLC patients [15]. Similarly, Wang et al. reported that mutations in fatty acid synthase were related to superior benefits from ICB in a large cohort of melanoma and NSCLC patients [16]. Furthermore, a study led by Yu et al. on lung squamous cell carcinoma observed that TP53 wildtype, especially when co-occurring with LRP1B wildtype, is associated with improved survival after anti-PD1 therapy [17]. It is anticipated that with the accumulation of genomic profiling data from patients who received ICB-based treatment, additional genomic features will emerge as potential predictive biomarkers in NSCLC patients with or without actionable mutations.

Biomarker-based therapeutic decision-making is the foundation of modern precision oncology. However, tissue-based tests often face limitations due to inadequate specimens [5,6,18,19,20] and intra-tumor heterogeneity [5,6,7,21]. Moreover, longitudinal tissue-based profiling is often not feasible in clinical practice. Radiological images contain rich information that reflects the anatomical and functional characteristics of the tumor and its microenvironment. However, these images’ complex anatomical and morphological features surpass the analytic capacity of human eyes. Therefore, artificial intelligence (AI), such as machine learning, has become a promising modality for extracting informative data from these intricate images [22]. Two radiogenomics studies applied machine learning approaches to predict oncodriver mutation status and PD-L1 level in this issue, showing promise. He et al. used a machine-learning approach to predict EGFR mutation status [23]. At the same time, Shao et al. developed a multi-label multi-task deep learning system for the same purpose to predict the mutation status of multiple oncodrivers and PD-L1 levels [24]. As the performance of machine learning depends heavily on sample size and data quantity, future studies with larger sample sizes and high-quality image/molecular data are expected to improve radiogenomic predictions further.

In addition to predicting therapeutic response, actionable mutations have also been used for prognostication. For example, in this Special Issue, Tian et al. reported the value of testing for oncodriver alterations in detecting occult metastatic disease in morphologically negative lymph nodes [25]. In contrast, Zhao et al. reported that oncogenic EFNA4 amplification may promote lymph node metastasis and be associated with poor prognosis in lung adenocarcinomas [26].

With ongoing efforts in molecular profiling of NSCLC, more actionable mutations are being discovered, and more actionable mutations are becoming targetable, such as the recent example of Kras G12C [27]. Furthermore, patients with NSCLC are subtyped into different molecular subgroups with varying response profiles based on co-mutations [28]. Therefore, it is reasonable to anticipate that most, if not all, NSCLC tumors will eventually be found to carry mutations that we can act on.
